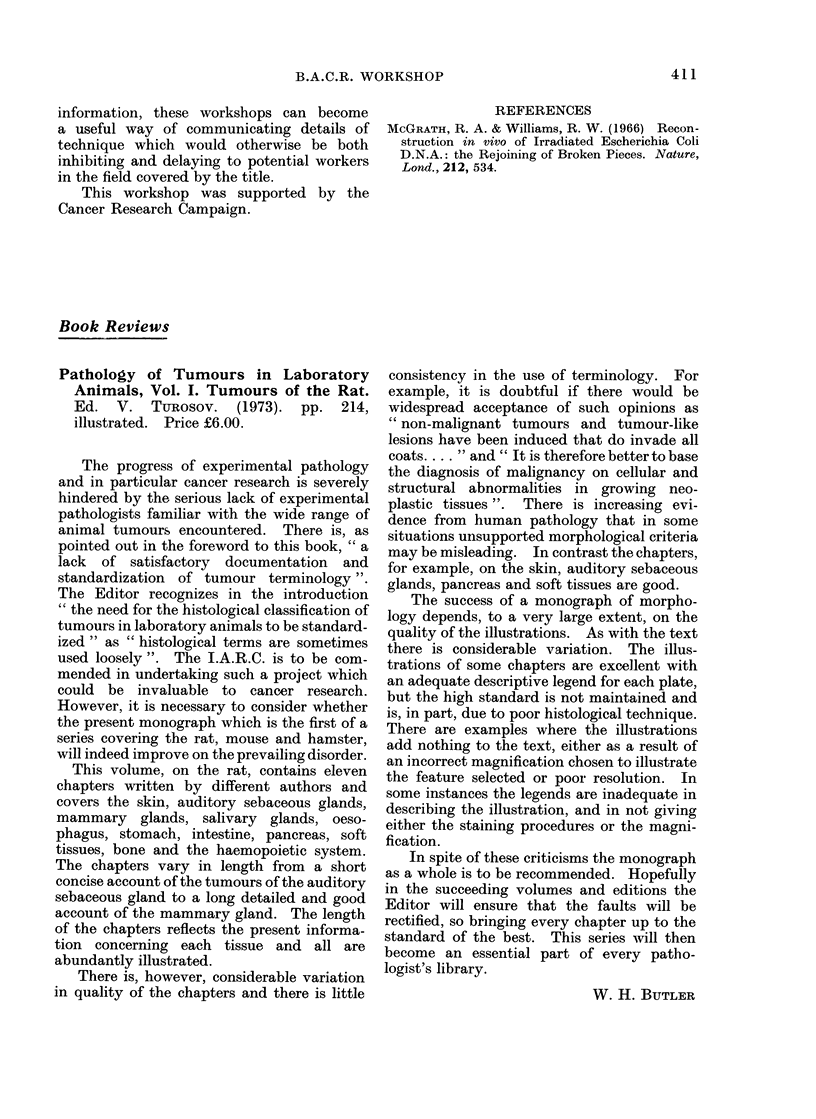# Pathology of Tumours in Laboratory Animals, Vol. I. Tumours of the Rat

**Published:** 1974-05

**Authors:** W. H. Butler


### *Book Reviews*

**Pathology of Tumours in Laboratory Animals, Vol. I. Tumours of the Rat.** Ed. V. Turosov. (1973). pp. 214, illustrated. Price £6.00.

The progress of experimental pathology and in particular cancer research is severely hindered by the serious lack of experimental pathologists familiar with the wide range of animal tumours encountered. There is, as pointed out in the foreword to this book, " a lack of satisfactory documentation and standardization of tumour terminology ". The Editor recognizes in the introduction " the need for the histological classification of tumours in laboratory animals to be standardized " as " histological terms are sometimes used loosely ". The I.A.R.C. is to be commended in undertaking such a project which could be invaluable to cancer research. However, it is necessary to consider whether the present monograph which is the first of a series covering the rat, mouse and hamster, will indeed improve on the prevailing disorder.

This volume, on the rat, contains eleven chapters written by different authors and covers the skin, auditory sebaceous glands, mammary glands, salivary glands, oesophagus, stomach, intestine, pancreas, soft tissues, bone and the haemopoietic system. The chapters vary in length from a short concise account of the tumours of the auditory sebaceous gland to a long detailed and good account of the mammary gland. The length of the chapters reflects the present information concerning each tissue and all are abundantly illustrated.

There is, however, considerable variation in quality of the chapters and there is little consistency in the use of terminology. For example, it is doubtful if there would be widespread acceptance of such opinions as " non-malignant tumours and tumour-like lesions have been induced that do invade all coats. . . . " and " It is therefore better to base the diagnosis of malignancy on cellular and structural abnormalities in growing neoplastic tissues ". There is increasing evidence from human pathology that in some situations unsupported morphological criteria may be misleading. In contrast the chapters, for example, on the skin, auditory sebaceous glands, pancreas and soft tissues are good.

The success of a monograph of morphology depends, to a very large extent, on the quality of the illustrations. As with the text there is considerable variation. The illustrations of some chapters are excellent with an adequate descriptive legend for each plate, but the high standard is not maintained and is, in part, due to poor histological technique. There are examples where the illustrations add nothing to the text, either as a result of an incorrect magnification chosen to illustrate the feature selected or poor resolution. In some instances the legends are inadequate in describing the illustration, and in not giving either the staining procedures or the magnification.

In spite of these criticisms the monograph as a whole is to be recommended. Hopefully in the succeeding volumes and editions the Editor will ensure that the faults will be rectified, so bringing every chapter up to the standard of the best. This series will then become an essential part of every pathologist's library.

W. H. Butler